# Genetic Determinants of Radiosensitivity: Evidence of Radioresistance-Associated SNP Enrichment in Occupational Workers Chronically Exposed to Low-Dose Radiation

**DOI:** 10.3390/genes17020191

**Published:** 2026-02-03

**Authors:** Dauren Botbayev, Kamalidin Sharipov, Ayaz Belkozhayev, Bakhytzhan Alzhanuly, Ulbossyn Yerkinbek, Daulet Sharipov, Alexandr Gulyayev, Sayagul Kairgeldina, Kanat Tekebayev, Gulnur Zhunussova, Madina Baurzhan

**Affiliations:** 1 Department of Chemical and Biochemical Engineering, Geology and Oil-Gas Business Institute Named After K. Turyssov, Satbayev University, Almaty 050043, Kazakhstan; a.belkozhayev@satbayev.university; 2 Structural and Functional Genomics Laboratory of M.A. Aitkhozhin Institute of Molecular Biology and Biochemistry, Almaty 050012, Kazakhstan; shkamalidin@gmail.com (K.S.); bakhytzhan.alzhanuly@gmail.com (B.A.); 3 Department of Biochemistry, Asfendiyarov Kazakh National Medical University, Almaty 050000, Kazakhstan; 4 Department of Biology, Faculty of Natural Sciences and Geography, Abai Kazakh National Pedagogical University, Almaty 050010, Kazakhstan; ulbosyn_e_1993@mail.ru; 5 National Laboratory Astana, Nazarbayev University, Astana 010000, Kazakhstan; daulet.sharipov@nu.edu.kz; 6 Ministry of Health of the Republic of Kazakhstan, Research Institute of Balneology and Medical Rehabilitation, Astana 010000, Kazakhstan; akin@mail.ru (A.G.); sanborovoe@mail.kz (S.K.); kanat_7@mail.ru (K.T.); 7 Laboratory of Drug Discovery and Development, Nazarbayev University, Astana 010000, Kazakhstan; 8 Laboratory of Molecular Genetics, Institute of Genetics and Physiology, Almaty 050060, Kazakhstan; gulnur_j@mail.ru

**Keywords:** radioresistance, *TP53*, p21, chronic radiation exposure, radiogenomics, DNA damage response

## Abstract

**Background**: Interindividual radiosensitivity is largely driven by genetic regulation of DNA damage recognition, repair, and cell-cycle control. *TP53* and *CDKN1A* (*p21*) are key genomic markers associated with differential responses to ionizing radiation. **Methods**: This study investigated eight functional SNP markers across several key genes involved in DNA damage responses and cellular stress regulation, including *TP53, CDKN1A/p21, APC, VEGF, XPD,* and *RAD51*, in occupational groups exposed to chronic low-dose ionizing radiation at the Stepnogorsk Mining Chemical Combine and the Balkashinskoye uranium deposit. Genotyping was performed using PCR-based assays followed by restriction fragment length polymorphism (RFLP) analysis. Allele and genotype frequencies were compared between radiation-exposed workers and matched controls within Kazakh and Russian ethnic subgroups. Statistical differences were assessed using χ^2^ tests, and associations with radioresistance were interpreted based on established functional characteristics of each polymorphism. **Results**: Four SNPs showed significant allele and genotype frequency shifts in radiation-exposed populations. The *TP53* intron 3 insertion allele, *TP53* intron 6 A allele, *TP53* Pro72 (C) allele, and p21 codon 31 A allele were consistently enriched among exposed individuals. The strongest deviations were observed in Russian workers from Stepnogorsk (*p* < 0.01). These alleles are functionally associated with enhanced DNA repair efficiency, modified apoptotic responses, and strengthened cell-cycle checkpoint regulation. **Conclusions**: Significant enrichment of radioresistance-associated *TP53* and *CDKN1A* (p21) variants was observed in uranium industry workers chronically exposed to low-to-moderate ionizing radiation. The observed patterns support a polygenic model of adaptive responses and emphasize the importance of genetic background in determining individual radiosensitivity under occupational exposure conditions.

## 1. Introduction

The development of the nuclear industry represents a key driver of modern scientific and technological advancement as nuclear energy offers a reliable, high-efficiency, and economically viable source capable of meeting the growing global demand for electricity. The expanding use of nuclear technologies contributes to industrial modernization, strengthens national energy security, and promotes sustainable socio-economic development worldwide. This issue is particularly relevant for Kazakhstan, which consistently ranks as the world’s leading producer of uranium. According to the World Nuclear Association, Kazakhstan produced approximately 23,270 tonnes of uranium in 2024 and possesses nearly 14% of the world’s identified recoverable uranium resources [1]. Given its dominant position in the global nuclear fuel cycle, Kazakhstan faces an increased demand for effective radiation safety measures and comprehensive radiological protection strategies to safeguard occupationally exposed populations and ensure long-term environmental and public health security.

Despite the substantial benefits associated with nuclear technologies, ionizing radiation presents significant health risks. The biological and medical consequences of radiation exposure vary considerably depending on the absorbed dose, dose rate, and individual physiological characteristics. High-dose exposures that exceed established threshold levels lead to deterministic (non-stochastic) effects such as acute radiation syndrome (ARS), hematopoietic suppression, gastrointestinal injury, and multi-organ failure. These effects exhibit a well-defined dose–response relationship, and their likelihood increases sharply once the threshold dose is surpassed [2].

In contrast, the effects of low-dose radiation exposure are probabilistic in nature. The long-term accumulation of small radiation doses may induce stochastic outcomes, including carcinogenesis and hereditary alterations. According to the widely accepted linear-no-threshold (LNT) model, even minimal exposures carry a measurable degree of risk, despite the absence of immediate clinical manifestations [2]. Although regulatory dose limits serve as an essential tool for risk reduction, they cannot ensure complete radiological safety because the probability of stochastic effects persists at any dose. This highlights the importance of comprehensive radiation protection strategies grounded in the ALARA principle (“as low as reasonably achievable”), as recommended by the International Commission on Radiological Protection [3].

Prolonged exposure to low doses of ionizing radiation is believed to induce subtle yet biologically meaningful alterations at the cellular and molecular levels. These effects include DNA damage, disturbances in genome integrity, activation of oncogenic signaling pathways, metabolic dysregulation, and impairment of hematopoietic function [2,3,4]. Importantly, susceptibility to such low-dose health effects varies considerably among individuals due to complex interactions between genetic predisposition, physiological status, lifestyle factors, and environmental conditions. As noted by Hall and Giaccia, inter-individual radiosensitivity represents a multifactorial phenomenon influenced by both inherited and acquired determinants [5]. This variability underscores one of the major unresolved challenges in modern radiobiology: the identification of reliable and mechanistically informative biomarkers capable of identifying individuals with heightened or reduced radiosensitivity.

Of particular interest is the assessment of the workers at Kazakhstan’s coal deposits, which contain naturally occurring radioactive elements (uranium, thorium, and potassium-40). Kazakhstan’s coals are generally characterized as slightly radioactive, but the natural radioactivity of Kazakhstan’s coals has been poorly studied. Average uranium and thorium contents in Kazakhstan’s coals are 1.8 and 2.2 g/t, respectively, and in ash, they are 8.7 and 10.6 g/t. It is known that the average uranium content in the studied coals of Northern Asia varies from 0.4–0.5 g/t (Karaganda and Torgai basins, Karazhyra deposit, Kazakhstan) to 32.8 g/t (Adun-Chulun deposit, Mongolia) [6]. 

It is known that during the combustion of coals, even those with low concentrations of radionuclides in the combustion waste (solid ash, slag, fly ash), the content of radionuclides (uranium-238 and its decay products, thorium-232 (and its decay products) and potassium-40) increases by 3–8 times compared to the original coal. Thus, in the ash and slag waste of the Toparskaya GRES, which burns Karaganda coal, the concentration factors of radionuclides vary in the range of 2.5–10.9. The obtained results indicate that during the combustion of weakly radioactive coals, a concentration of radionuclides in the ash and slag waste occurs [7,8,9]. 

Radiation genetics has emerged as a key discipline addressing interindividual variability in biological responses to ionizing radiation. Inherited genetic factors are considered among the most promising determinants of radiation-associated health risks, as they influence DNA repair capacity, apoptosis regulation, oxidative stress tolerance, and cell-cycle control [10]. Genetic variation plays a central role in the development of multifactorial diseases, including immune-mediated disorders, cardiovascular diseases, and cancer, underscoring the broader relevance of human genomic diversity [11]. Identifying genetic markers associated with increased susceptibility to radiation-related conditions may therefore enable the prevention of unnecessary exposure among radiosensitive individuals and support more precise diagnostic and therapeutic decision-making in radiology and radiotherapy [8].

Advances in molecular genetics, particularly those enabled by the completion of the Human Genome Project, have substantially enhanced the capacity to investigate genetic determinants of radiosensitivity [12]. One major outcome of the project is the ability to characterize human genetic diversity through single nucleotide polymorphisms (SNPs), the most common form of genomic variation. SNP-based analyses provide powerful tools for identifying gene variants directly or indirectly associated with individual biological responses to environmental stressors, including ionizing radiation [13]. This genomic approach offers a strong methodological framework for exploring inherited susceptibilities and improving the understanding of inter-individual differences in radiation response, thereby supporting the development of personalized radiological protection and precision radiotherapy strategies [14].

Among the genetic systems investigated to date, some of the strongest inter-individual variability has been documented in genes responsible for maintaining genome integrity and regulating the cell cycle. These include key components of the DNA repair, apoptosis, and cellular stress-response pathways such as *TP53*, *CDKN1A*/p21, *APC*, *VEGF*, *XPD*, and *RAD51*. Variability within these genes is of particular interest because they play central roles in recognizing and repairing radiation-induced DNA lesions, coordinating cell-cycle arrest, and determining cell fate decisions following genotoxic stress [15]. Taken together, these molecular systems represent a highly promising framework for elucidating the genetic mechanisms underlying radiosensitivity and radioresistance [16].

## 2. Materials and Methods

### 2.1. Study Population and Sample Collection

This study involved male participants of Kazakh and Russian ethnicity with no self-reported personal or family history of cancer or hereditary disorders. Venous blood samples were obtained from workers employed at two uranium-mining enterprises in Kazakhstan. The first occupational group included 238 workers from the Balkhashinskoe uranium deposit in Shantobe (54 Kazakhs, 184 Russians). The second group comprised 224 workers from the Stepnogorsk Mining and Chemical Combine (52 Kazakhs, 172 Russians).

A control cohort consisting of 289 healthy male donors (129 Kazakhs, 160 Russians) was recruited from the Almaty Blood Center. Control individuals had no occupational radiation exposure and no known chronic illnesses at the time of sampling. Only male participants were included to reduce sex-related variability in radiosensitivity, hormonal status, and genetic background. The overall distribution of participants across study groups and ethnicities is summarized in Table 1.

Occupational exposure at the studied enterprises (primarily in situ recovery mining and hydrometallurgical processing) is generally low. According to recent Kazatomprom Integrated Annual Reports, the average annual effective radiation dose for Group A personnel (occupationally exposed workers) ranged from 1.36 to 1.51 mSv/year (including a natural background contribution of approximately 0.7–1.2 mSv/year), with maximum individual doses typically not exceeding 4–6 mSv/year and remaining well below the regulatory limit of 20 mSv/year [1,2]. Historical and site-specific data, including urine bioassays and electron paramagnetic resonance (EPR) tooth enamel dosimetry, indicate that cumulative career doses for workers with 10–20+ years of employment often stay in the low range (<100 mSv), although individual variability exists due to internal exposure pathways (radon progeny and uranium dust) [17,18]. 

Peripheral venous blood (5–10 mL) was collected into EDTA-coated tubes under standardized conditions. All samples were anonymized, coded, and transported on ice to the M.A. Aitkhozhin Institute of Molecular Biology and Biochemistry where genomic DNA was extracted using certified molecular-genetic protocols.

### 2.2. DNA Extraction

Genomic DNA was extracted using the QIAamp DNA Mini Kit (Qiagen, Hilden, Germany) according to the manufacturer’s protocol [19]. Whole-blood samples were lysed with Buffer AL and proteinase K, followed by ethanol addition and purification using DNeasy Mini silica spin columns. Wash steps were performed with Buffers AW1 and AW2, and DNA was eluted in Buffer AE. All centrifugation steps were conducted at room temperature (15–25 °C). To maximize yield, the elution step was performed twice. Extracted DNA was stored at −20 °C until genotyping.

### 2.3. PCR Amplification and Genotyping

Primers were designed using Primer3 (version 0.4.0) and Primer Express software (Version 3.0) [20], considering GC content, melting temperature (Tm), and potential secondary structures. PCR amplification was performed using Taq DNA Polymerase (Thermo Fisher Scientific, Waltham, MA, USA) in 20 µL reaction volumes containing 50 ng of genomic DNA. Primer sequences and PCR conditions are shown in Table 2. Amplification products were separated on 2% agarose gels.

### 2.4. RFLP Analysis

Restriction fragment length polymorphism (RFLP) genotyping was performed for loci requiring enzymatic digestion. Genotyping call rates were >98% for all SNPs (98.2–99.7%); failed calls were excluded due to lack of SNP specificity. Restriction endonucleases (Thermo Fisher Scientific, USA) were used according to the manufacturer’s instructions. Restriction enzymes and expected fragment sizes are shown in Table 3.

### 2.5. Gel Electrophoresis and Imaging

PCR products were resolved on 2% agarose gels prepared in 1× TAE buffer and stained with ethidium bromide. Electrophoresis was performed at 100 V for 1 h. DNA fragments were visualized under UV illumination using a Bio-Rad GelDoc XR+ documentation system, and images were analyzed using Image Lab Software v6.0 (Bio-Rad Laboratories, Hercules, CA, USA) [21].

### 2.6. Statistical Analysis

Statistical analyses were performed using Statistica v5.0 and Statistica 2005 (StatSoft Inc., Tulsa, OK, USA) [22]. Allele and genotype frequencies were compared using Pearson’s χ^2^ test. A significance threshold of *p* < 0.05 was used. Odds ratios (ORs) and 95% confidence intervals (CIs) were calculated to assess associations between genotypes and outcomes. All loci were tested for Hardy–Weinberg equilibrium (HWE). Logistic regression was applied to evaluate independent associations between genetic variants and phenotypic parameters.

## 3. Results

### 3.1. Distribution of Candidate SNP Markers and Their Association with Radioresistance

**Figure 1:**
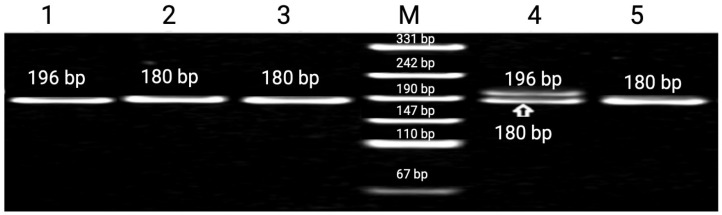
Typical 8% polyacrylamide gel electrophoresis of PCR products of the TP53 intron 3 (rs17878362) polymorphism. Lane M—GeneRuler™ 100 bp DNA Ladder (Thermo Fisher Scientific, USA) used as a molecular size marker. Lane 1—homozygous mutant genotype (with 16 bp insertion); Lanes 2, 3, and 5—homozygous wild-type genotype (no insertion); Lane 4—heterozygous genotype.

A total of eight SNP markers were analyzed, of which, four polymorphisms *TP53* intron 3 (rs17878362), *TP53* intron 6 (rs1625895), *TP53* exon 4 Arg72Pro (rs1042522), and *CDKN1A*/p21 codon 31 (rs1801270) demonstrated statistically significant differences in allele and genotype frequencies between radiation-exposed workers and control groups. The results for *APC*, *VEGF*, *XPD*, and *RAD51* polymorphisms are presented in Supplementary Tables S1 and S2. Functional annotation of these variants suggests their involvement in major DNA damage response pathways. Specifically, the *TP53* intron 3 insertion, *TP53* intron 6 A allele, *TP53* Pro allele, and p21 codon 31 A allele are associated with enhanced DNA repair, modified apoptosis signaling, improved checkpoint activation, and ultimately increased resistance to chronic low-dose ionizing radiation [23,24,25]. A representative electrophoretic pattern of the *TP53* intron 3 (rs17878362) polymorphism is shown in Figure 1.

The distribution of genotypes and allele frequencies of the *TP53* intron 3 (rs17878362) polymorphism was analyzed in uranium-mine workers and healthy control individuals. Comparative analysis revealed differences in genotype and allele frequencies between the two groups, reflecting potential associations between this polymorphism and occupational exposure to ionizing radiation. Detailed genotype distributions and allele frequencies are presented in Table 4.

Analysis of the *TP53* intron 3 insertion/deletion polymorphism revealed notable differences between exposed and unexposed individuals. Among the Russian ethnic group working at the Stepnogorsk Mining and Chemical Combine, the frequency of the insertion allele was significantly higher compared to the control cohort (*p* = 0.015). This redistribution implies potential adaptive allele enrichment in populations subjected to prolonged radiation exposure (Table 4). The insertion variant may contribute to mRNA structural stability and altered splicing, which is potentially relevant to modulating p53 responses under occupational genotoxic stress [26,27].

### 3.2. TP53 Intron 6 Polymorphism (rs1625895)

**Figure 2:**
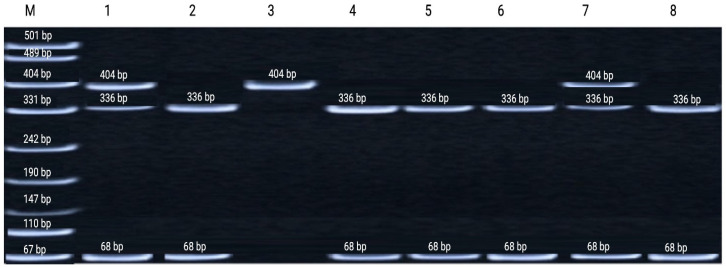
Representative electrophoresis of restriction fragments of the amplified TP53 intron 6 (rs1625895) region on 8% polyacrylamide gel. Lane M—GeneRuler™ 100 bp DNA Ladder (Thermo Fisher Scientific, USA) used as a molecular size marker; Lanes 2, 4, 5, 6, 8—homozygous wild-type genotype (no mutation in the restriction site); Lanes 1, 7—heterozygous genotype (mutation present in only one DNA strand); Lane 3—homozygous mutant genotype.

The rs1625895 (G>A) polymorphism exhibited ethnicity-specific differences across exposed populations. In the Kazakh group, genotype frequencies differed significantly between irradiated workers and the control sample (*p* = 0.003). In the Russian subgroup from Stepnogorsk, both genotype (*p* = 0.0001) and allele frequencies (*p* = 0.002) showed highly significant deviations. These findings are consistent with patterns observed in occupationally exposed groups. Such enrichment aligns with the functional role of the variant in modulating intronic regulatory elements and fine-tuning *TP53* transcriptional activity [28]. A representative electrophoretic pattern of restriction fragments for the *TP53* intron 6 (rs1625895) polymorphism is shown in Figure 2.

The distribution of genotypes and allele frequencies of the *TP53* intron 6 (rs1625895) polymorphism was analyzed in uranium-mine workers and healthy control individuals. Significant differences were observed between exposed and control groups, particularly with enrichment of the A allele among irradiated workers. These findings support the potential role of this variant in modulating individual responses to chronic low-dose ionizing radiation. Detailed genotype and allele frequency distributions are presented in Table 5.

### 3.3. TP53 Exon 4 Arg72Pro Polymorphism (rs1042522)

**Figure 3:**
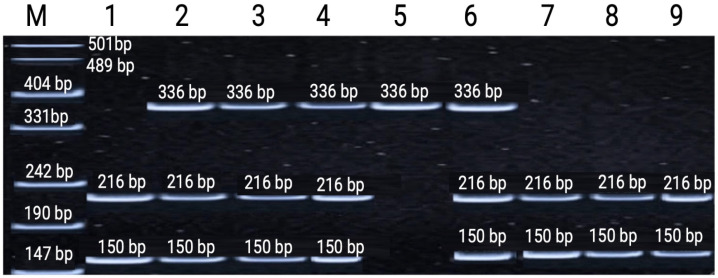
Representative polyacrylamide gel electrophoresis of restriction fragments of the TP53 exon 4 (Arg72Pro, rs1042522) amplicon. Lane M—GeneRuler™ 100 bp DNA Ladder (Thermo Fisher Scientific, USA), used as a molecular size marker; Lanes 1, 7, 8, and 9—homozygous wild-type genotype (Arg/Arg); Lanes 2, 3, 4, and 6—heterozygous genotype (Arg/Pro); Lane 5—homozygous mutant genotype (Pro/Pro).

The *TP53* exon 4 Arg72Pro (rs1042522) polymorphism showed significant differences in some, but not all, subgroups. In the Balkhashinskoye (Shantobe) cohort, allele frequencies differed significantly among Kazakhs (*p* = 0.006) and both genotype (*p* = 0.019) and allele (*p* = 0.001) frequencies differed among Russians. In the Stepnogorsk cohort, genotype differences reached nominal significance only among Kazakhs (*p* = 0.048), while allele comparison and Russian subgroup results were non-significant (all *p* > 0.10). Overall, a higher frequency of the Pro (C) allele and Pro/Pro genotype was observed in several exposed subgroups, particularly at the Balkhashinskoye site. The Pro72 variant has been previously reported to exhibit reduced mitochondrial apoptotic activity compared to Arg72 [29], which may plausibly contribute to differences in cellular responses under chronic low-dose exposure—although this mechanism was not directly tested in the present study. Representative restriction fragment patterns are shown in Figure 3.

The distribution of genotypes and allele frequencies of the *TP53* exon 4 Arg72Pro (rs1042522) polymorphism was analyzed in uranium-mine workers and healthy control individuals. Significant differences in both genotype and allele frequencies were observed between exposed and control groups, with a higher prevalence of the Pro (C) allele among workers. These results support a potential role for this polymorphism in modulating cellular apoptotic responses under chronic low-dose radiation exposure. Detailed genotype and allele frequency distributions are presented in Table 6.

### 3.4. p21 (CDKN1A) Codon 31 Polymorphism (rs1801270)

**Figure 4:**
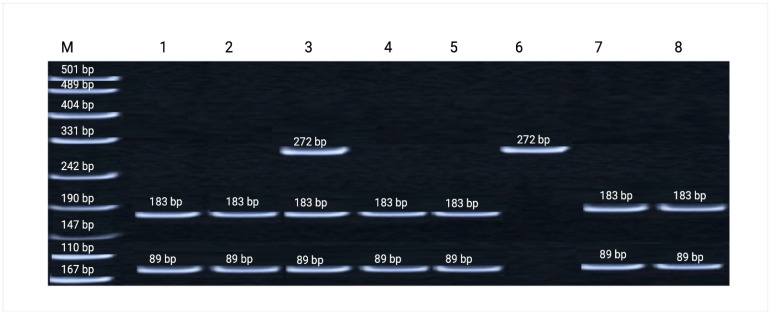
Representative polyacrylamide gel electrophoresis of restriction fragments of the amplified *CDKN1A* (p21) codon 31 (rs1801270) regions. Lane M—GeneRuler™ 100 bp DNA Ladder (Thermo Fisher Scientific, USA), used as a molecular size marker; Lanes 1, 2, 4, 5, 7, 8—homozygous wild-type genotype (Ser/Ser); Lane 6—homozygous mutant genotype (Arg/Arg); Lane 3—heterozygous genotype (Ser/Arg).

The analysis of the p21 codon 31 variant revealed a consistent trend toward a higher frequency of the A allele in radiation-exposed groups. This allele is known to enhance G1/S checkpoint control and facilitate the repair of radiation-induced DNA damage, thereby contributing to radioresistance. Although the magnitude of the differences varied across ethnic groups, the general pattern supports the functional contribution of this variant to interindividual variability in radiosensitivity [30,31]. Representative restriction fragment patterns of the *CDKN1A* (p21) codon 31 amplicon are shown in Figure 4.

The distribution of genotypes and allele frequencies of the *CDKN1A*/p21 codon 31 (rs1801270) polymorphism was analyzed in uranium-mine workers and healthy control individuals. A higher frequency of the A allele was observed among exposed workers, consistent with its reported role in enhancing DNA damage repair and promoting radioresistance. These findings suggest that this variant may contribute to individual differences in cellular responses to chronic low-dose radiation. Detailed genotype and allele frequency distributions are presented in Table 7.

## 4. Discussion

The present study provides substantial evidence that genetic polymorphisms within TP53 and *CDKN1A* (p21) contribute to interindividual differences in susceptibility to chronic low-dose ionizing radiation. The significant allele frequency shifts observed in workers from the Stepnogorsk Mining and Chemical Combine and the Balkashinskoye uranium deposit may reflect genetic differences potentially related to occupational exposures. 

Observation of multiple radioresistance-associated alleles such as *TP53* intron 3 insertion, *TP53* intron 6 A allele, *TP53* Pro72, and the *p21* codon 31 A allele suggests a polygenic model of adaptation, consistent with previous reports indicating that radiosensitivity is regulated by several genes rather than single-locus variation [32,33]. These findings align with a polygenic model of variability in DNA damage response described by Hall et al. [5], in which efficient DNA repair, enhanced checkpoint control, and reduced apoptotic loss may contribute to differences in cellular responses under occupational exposure.

The significant enrichment of the *TP53* intron 3 insertion allele (rs17878362) in exposed individuals is consistent with earlier research showing that this polymorphism affects *TP53* mRNA processing and alternative splicing [34]. Increased expression of certain *TP53* isoforms (e.g., Δ40p53) may modulate apoptosis and cell-cycle arrest under chronic radiation exposure, supporting cellular survival [35].

Similarly, the observed enrichment of the *TP53* intron 6 A allele (rs1625895) corresponds with findings indicating that intronic variants alter transcription factor binding sites and *TP53* regulatory dynamics [36,37]. Intronic regions are increasingly recognized as contributors to radiation response, which act via modulation of chromatin structure and co-factor binding [38].

The Pro allele (C) of the *TP53* Arg72Pro polymorphism, which was found at a higher frequency among radiation-exposed workers, is functionally characterized as reducing p53-mediated apoptosis and the shift toward cell-cycle arrest and DNA repair pathways. This allele has been shown to diminish mitochondrial translocation of p53 and weaken its apoptotic activity while enhancing G1/S checkpoint control and promoting cell survival under stress conditions [39]. Such a functional profile may contribute to cellular survival under occupational genotoxic stress, where excessive apoptosis could compromise tissue homeostasis.

Dumont et al. demonstrated that the Arg72 variant induces apoptosis up to five times more efficiently than the Pro72 variant, indicating substantially different functional capacities between these isoforms. Consequently, individuals carrying the Pro/Pro genotype may exhibit greater resilience under long-term genotoxic exposure due to preferential activation of cell-cycle arrest and DNA repair mechanisms rather than apoptosis-driven cell loss. The *p21* codon 31 A allele was also enriched in exposed individuals and is associated with increased protein stability and enhanced G1/S checkpoint activation [10]. Keshava et al. demonstrated that this variant strengthens cell-cycle arrest following DNA damage [36], thereby reducing the likelihood of replication of damaged DNA. Such properties align closely with the adaptive needs of populations experiencing continuous low-dose radiation.

The pronounced allele frequency differences between Kazakh and Russian subgroups emphasize the necessity of considering population genetic structure in radiosensitivity research. Similar ethnicity-dependent effects have been documented in studies on *TP53* and *CDKN1A* polymorphisms in East European and Central Asian populations [40,41]. Such background variation may modulate the magnitude and direction of environmental selection pressures.

The concordant enrichment of radioresistant alleles across multiple loci suggests that occupational exposures in the uranium sector may contribute to observed patterns in long-tenured workers. Although evolutionary timescales are short, strong environmental stressors can yield measurable allele frequency shifts within a few generations or even within survivor cohorts [42]. Survivorship bias may also contribute: individuals with radiosensitive genotypes may have been more susceptible to health complications, leaving a genetically enriched group of survivors, as was observed in studies of Chernobyl cleanup workers and radiology personnel [43,44].

The observed allele frequency shifts must be interpreted cautiously in the context of a cross-sectional study design. A cross-sectional comparison cannot distinguish between potential evolutionary processes (e.g., selection over generations) and non-evolutionary explanations. Key alternatives include:

Survivorship (healthy worker survivor) bias: Workers with longer employment durations (mean 11–17 years) represent a surviving cohort. Individuals with genotypes conferring higher susceptibility to radiation-related health effects (or other occupational stressors) may have left employment earlier due to illness or other reasons, leading to relative enrichment of “resistant” alleles among remaining long-tenured workers. Similar survivor bias has been documented in occupational radiation cohorts, such as uranium miners and Chernobyl liquidators, where healthier individuals persist in high-exposure roles [45,46,47].

Population stratification: The study included Kazakh and Russian ethnic subgroups with known genetic differences (e.g., distinct ancestry components in Central Asian vs. Slavic populations). Although we stratified analyses by ethnicity, residual stratification or unaccounted substructures could contribute to allele frequency differences unrelated to exposure [48,49]. This is particularly relevant in Kazakhstan, where ethnic groups show admixture patterns and varying historical migration influences.

These factors, combined with other occupational confounders (e.g., dust, chemicals, lifestyle), provide plausible non-selection explanations for the patterns. We have explicitly stated that the findings represent associations in exposed vs. unexposed groups, not evidence of radiation-driven selection within the study timeframe. Future longitudinal or family-based studies would be needed to explore transgenerational or longer-term effects.

The molecular patterns identified in this study underscore the importance of integrating genetic screening into occupational radiation safety programs. A growing body of evidence supports the use of *TP53*, *CDKN1A*, *ATM*, and *XRCC* family polymorphisms as predictive biomarkers for individualized radiation risk assessment [50,51,52]. Our findings contribute valuable data for Central Asian populations, which remain understudied in global radiogenomic research. 

In addition, in the future, we plan to expand the field of research to include the population of coal miners in Kazakhstan exposed to coal dust from low-radioactive types (rocks) of coal.

While the associations identified are compelling, several limitations should be acknowledged. A key limitation is the lack of individual or group-specific dosimetry data for the recruited workers, which precludes direct dose–response analysis and causal attribution of allele frequency shifts to ionizing radiation exposure. Aggregate data from Kazatomprom indicate predominantly low annual doses (1.36–1.51 mSv/year on average for Group A personnel), but these do not replace cohort-specific measurements and may not fully capture historical or internal exposure variability (e.g., radon progeny, uranium dust). Future research should incorporate urine bioassays and electron paramagnetic resonance (EPR) tooth enamel dosimetry to enable precise exposure assessment and mechanistic studies. 

Additional limitations include moderate sample sizes for some subpopulations and the absence of functional cellular assays to validate mechanistic hypotheses. Future studies should integrate genome-wide approaches, transcriptomic profiling, DNA repair assays, and long-term health outcome monitoring to better characterize genotype–phenotype correlations. Machine learning models integrating multi-SNP profiles could further enhance the prediction accuracy for individual radiosensitivity. 

## 5. Conclusions

This study demonstrates that polymorphisms within *TP53* and *CDKN1A* (p21), along with other DNA damage-response genes, are significantly associated with inter individual differences in susceptibility to chronic low-dose ionizing radiation. The consistent enrichment of variants previously associated in the literature with efficient DNA repair and altered cell fate decisions is consistent with a polygenic model of variability in DNA damage response and underscores the importance of genetic background in shaping individual radiosensitivity. These associations represent hypotheses that require functional validation through cellular assays, reporter systems, or gene-editing approaches in future studies. These findings underscore the potential value of incorporating molecular-genetic markers into occupational radiation protection and risk-stratification frameworks. Further research integrating functional assays and genome-wide analyses will be essential for validating these markers and advancing personalized radiological safety strategies.

## Figures and Tables

**Table 1:** Characteristics of the study groups.

Study Group	Total Number of Samples	Kazakhs (n)	Russians (n)	Mean Age (Kazakhs), Years	Mean Work Experience (Kazakhs), Years	Mean Age (Russians), Years	Mean Work Experience (Russians), Years
Control group	289	129	160	47.7	–	42.1	–
Balkhashinskoe	238	54	184	44.0	11.0	49.0	13.7
Stepnogorsk	224	52	172	35.0	15.5	40.0	17.3

**Table 2:** Primer sequences and PCR cycling conditions.

Gene Segment	Forward Primer (5′→3′)	Reverse Primer (5′→3′)	Initial Denaturation	Cycling Conditions	Final Extension
*TP53* rs17878362	GGGACTGACTTTCTGCTCTT	TCAAATCATCCATTGCTTGG	95 °C, 10 min	40 cycles: 95 °C 1 min, 55 °C 1 min, 72 °C 1 min	72 °C, 10 min
*TP53* rs1625895	TGGCCATCTACAAGCAGTCA	TTGCACATCTCATGGGGTTA	94 °C, 1 min	30 cycles with stepwise annealing (60/58/56 °C)	72 °C, 10 min
*TP53* rs1042522	GGTAAGGACAAGGGTTGG	ACTGACCGTGCAAGTCACAG	95 °C, 5 min	35 cycles: 95 °C 30 s, 58 °C 40 s, 72 °C 40 s	72 °C, 5 min
*APC* exon 11	GGACTACAGGCCATTGCAGAA	GGCTACTCTCCAAAAGTCAA	95 °C, 6 min	35 cycles: 95 °C 1 min, 58 °C 30 s, 72 °C 30 s	72 °C, 5 min
*p21* rs1801270	GTCAGAACCGGCTGGGGATG	CTCCTCCCAACTCATCCCGG	94 °C, 5 min	35 cycles: 94 °C 30 s, 57.2 °C 30 s, 72 °C 30 s	72 °C, 7 min
*VEGF* −2549	GCTGAGAGTGGGGCTGACTAGGTA	GTTTCTGACCTGGCTATTTCCAGG	95 °C, 6 min	35 cycles: 94 °C 1 min, 57 °C 1.5 min, 72 °C 2 min	72 °C, 10 min
*RAD51* rs1801320	AGAGACCGAGCCCTAAGGA	CGCCTCACACACTCACCTC	95 °C, 3 min	35 cycles: 94 °C 30 s, 60.5 °C 30 s, 72 °C 1.5 min	72 °C, 5 min
*XPD* rs13181	ATCCTGTCCCTACTGGCCATTC	TGTGGACGTGACAGTGACAAAT	95 °C, 5 min	35 cycles: 94 °C 30 s, 64 °C 30 s, 72 °C 30 s	72 °C, 3 min

**Table 3:** Restriction enzymes and expected fragment sizes.

Gene and Locus	Restriction Enzyme	Fragment Sizes (bp)	Restriction Required
*TP53* exon 4	BstuI	165, 231, 396	Yes
*TP53* intron 6	MspI	68, 336, 404	Yes
*APC* exon 11	RsaI	133, 87, 46	Yes
*p21* codon 31	BlpI	272, 183, 89	Yes
*TP53* intron 3	None	180, 196	No
*VEGF* −2549	None	221, 229	No
*RAD51* rs1801320	Bst2UI	118, 113, 231	Yes
*XPD* rs13181	PstI	222, 158, 102, 64	Yes

**Table 4:** Distribution of genotypes and allele frequencies of the rs17878362 polymorphism among uranium-mine workers and healthy individuals.

Location	Population Group	Genotype	Miners	Controls	OR (95% CI)	95% CI	χ^2^ (Genotype)	*p* (Genotype)	χ^2^ (Allele)	*p* (Allele)
Stepnogorsk Mining and Chemical Combine	Kazakhs	I−/I−	0.750	0.756	1.391	0.455–2.056	0.554	0.456	0.291	0.589
		I−/I+	0.250	0.193						
		I+/I+	0.000	0.050						
	Russians	I−/I−	0.559	0.758	1.711	1.105–2.646	16.55	4.736	5.886	0.015
		I−/I+	0.429	0.192						
		I+/I+	0.011	0.050						
Balkashinskoye (Shantobe)	Kazakhs	I−/I−	0.709	0.829	1.876	0.959–3.670	3.086	0.078	3.404	0.065
		I−/I+	0.273	0.163						
		I+/I+	0.018	0.008						
	Russians	I−/I−	0.708	0.75	1.034	0.660–1.621	1.835	0.175	0.027	0.869
		I−/I+	0.276	0.202						
		I+/I+	0.016	0.048						

Footnote: I− = allele without the 16-bp insertion (wild-type/deletion allele); I+ = allele with the 16-bp insertion (duplication/insertion allele).

**Table 5:** Distribution of genotypes and allele frequencies of the rs1625895 polymorphism among uranium-mine workers and healthy individuals.

Location	Population Group	Genotype	Miners	Controls	OR (95% CI)	95% CI	χ^2^ (Genotype)	*p* (Genotype)	χ^2^ (Allele)	*p* (Allele)
Stepnogorsk Mining and Chemical Combine	Kazakh	GG	0.385	0.186	0.328	0.162–0.662	8.642	0.003	2.201	0.137
		GA	0.577	0.806						
		AA	0.038	0.008						
	Russian	GG	0.559	0.765	0.391	0.233–0.655	13.86	0.0001	9.395	0.002
		GA	0.429	0.218						
		AA	0.011	0.017						
Balkashinskoye (Shantobe)	Kazakh	GG	0.727	0.806	1.519	0.779–2.960	1.183	0.276	1.522	0.217
		GA	0.255	0.186						
		AA	0.018	0.008						
	Russian	GG	0.746	0.771	1.083	0.669–1.752	0.328	0.566	0.118	0.731
		GA	0.243	0.214						
		AA	0.011	0.016						

**Table 6:** Distribution of genotypes and allele frequencies of the rs1042522 polymorphism among uranium-mine workers and healthy individuals.

Location	Population Group	Genotype	Miners	Controls	OR (95% CI)	95% CI	χ^2^ (Genotype)	*p* (Genotype)	χ^2^ (Allele)	*p* (Allele)
Stepnogorsk Mining and Chemical Combine	Kazakh	AA	0.385	0.528	1.188	0.731–1.934	6.058	0.048	0.488	0.491
		AP	0.558	0.346						
		PP	0.058	0.126						
	Russian	AA	0.408	0.512	0.656	0.411–1.047	3.621	0.163	1.632	0.212
		AP	0.489	0.380						
		PP	0.103	0.107						
Balkashinskoye (Shantobe)	Kazakh	AA	0.354	0.527	1.867	0.323–0.835	4.264	0.118	7.411	0.006
		AP	0.146	0.124						
		PP	0.500	0.349						
	Russian	AA	0.399	0.504	1.901	1.190–3.036	7.886	0.019	10.07	0.001
		AP	0.071	0.124						
		PP	0.530	0.372						

**Table 7:** Distribution of genotypes and allele frequencies of the rs1801270 polymorphism among uranium-mine workers and healthy individuals.

Location	Population Group	Genotype	Miners	Controls	OR (95% CI)	95% CI	χ^2^ (Genotype)	*p* (Genotype)	χ^2^ (Allele)	*p* (Allele)
Stepnogorsk Mining and Chemical Combine	Kazakh	CC	0.588	0.500	1.429	0.740–2.758	3.171	0.074	5.641	0.012
		CA	0.294	0.468						
		AA	0.118	0.032						
	Russian	CC	0.715	0.683	1.165	0.731–1.859	1.043	0.302	1.053	0.301
		CA	0.244	0.299						
		AA	0.041	0.018						
Balkashinskoye (Shantobe)	Kazakh	CA	0.636	0.500	1.750	0.236–0.919	4.221	0.041	0.925	0.336
		CC	0.291	0.468						
		AA	0.073	0.032						
	Russian	CA	0.148	0.294	1.351	0.993–1.837	10.910	0.009	3.677	0.055
		CC	0.836	0.688						
		AA	0.016	0.019						

## Data Availability

The data presented in this study are available on request from the corresponding author.

## Supplementary Materials

The following supporting information can be downloaded at: https://www.mdpi.com/article/10.3390/genes17020191/s1, Table S1: Allele and Genotype Frequency Distribution of Selected Genes in Individuals Associated with the Stepnogorsk Mining and Chemical Combine. Table S2: Allele and Genotype Frequency Distribution of Selected Genes in Individuals Associated with Balkashinskoye (Shantobe).

## Abbreviations

ALARA	As Low As Reasonably Achievable
ARS	Acute Radiation Syndrome
HWE	Hardy–Weinberg Equilibrium
ICRP	International Commission on Radiological Protection
LNT	Linear No-Threshold
PCR	Polymerase Chain Reaction
RFLP	Restriction Fragment Length Polymorphism
SNP	Single-Nucleotide Polymorphism
