# Supplementary material for: Genetic Determinants of Radiosensitivity: Evidence of Radioresistance-Associated SNP Enrichment in Occupational Workers Chronically Exposed to Low-Dose Radiation
Source: Genes (Basel). 2026 Feb 3;17(2):191. doi: 10.3390/genes17020191 (PMC12940297; doi:10.3390/genes17020191)
Supplement: Supplementary file 1 [file genes-17-00191-s001.zip › genes-4118374-supplementary.pdf]

genes

MDPI

Supplementary Materials to

Article

Genetic Determinants of Radiosensitivity: Evidence of Radio-resistance-Associated SNP Enrichment in Occupational Workers Chronically Exposed to Low-Dose Radiation

Dauren Botbayev [1,2]*, Kamalidin Sharipov [2,3], Ayaz Belkozhayev [1,2], Bakhytzhan Alzhanuly [2], Ulbossyn Yerkinbek [4], Daulet Sharipov [5], Alexandr Gulyayev [6,7], Sayagul Kairgeldina [6], Kanat Tekebayev [6], Gulnur Zhunussova[8], Madina Baurzhan [6]*

[1] Department of Chemical and Biochemical Engineering, Geology and Oil-Gas Business Institute Named After K. Turyssov, Satbayev University, Almaty 050043, Kazakhstan; d.botbayev@satbayev.university, a.belkozhayev@satbayev.university

[2] Structural and Functional Genomics Laboratory of M.A. Aitkhozhin Institute of Molecular Biology and Biochemistry, Almaty 050012, Kazakhstan; shkamalidin@gmail.com, bakhytzhan.alzhanuly@gmail.com

[3] Department of Biochemistry, Asfendiyarov Kazakh National Medical University, Almaty 050000, Kazakhstan

[4] Department of Biology, Faculty of Natural Sciences and Geography, Abai Kazakh National Pedagogical University, Almaty 050010, Kazakhstan; ulbosyn_e_1993@mail.ru

[5] National Laboratory Astana, Nazarbayev University, Astana 010000, Kazakhstan; daulet.sharipov@nu.edu.kz

[6] Ministry of Health of the Republic of Kazakhstan, Research Institute of Balneology and Medical Rehabilitation, Astana 010000, Kazakhstan; akin@mail.ru, sanborovoe@mail.kz , kanat_7@mail.ru, madina_baurzhan@mail.ru

[7] Laboratory of Drug Discovery and Development, Nazarbayev University, Astana 010000, Kazakhstan

[8] Laboratory of Molecular Genetics, Institute of Genetics and Physiology, Almaty 050060, Kazakhstan; gulnur_j@mail.ru,

*Correspondence: d.botbayev@satbayev.university (DB), madina_baurzhan@mail.ru (MB)

Table S1. Allele and Genotype Frequency Distribution of Selected Genes in Individuals Associated with the Stepnogorsk Mining-Chemical Combine

Polymorphism	Population Group	Genotype	Miners	Controls	OR (95% CI)	CI, (95%)	χ^2 (Genotype)	p (Genotype)	χ^2 (Allele)	p (Allele)
APC	Kazakhs	CC	0,250	0,305	2,181	0,366-1,581	3,825	0,147	0,425	0,514
		CT	0,692	0,508						
		TT	0,058	0,188						
	Russians	CC	0,266	0,275	0,953	0,566-1,605	4,687	0,095	0,814	0,366
		CT	0,627	0,533						
		TT	0,107	0,192						
VEGF	Kazakh	DD	0,442	0,419	0,781	0,415-1,501	0,696	0,705	0,014	0,903
		DI	0,365	0,426						
		II	0,192	0,155						
	Russian	DD	0,285	0,272	0,735	0,474-1,121	2,556	0,278	0,431	0,511
		DI	0,453	0,531						
		II	0,262	0,198						

 https://doi.org/10.3390/genes17020191

rs1801320	Kazakh	GG	0,865	0,822						
		GC	0,115	0,171	1,395	0,552-3,481	0,291	0,582	0,231	0,621
		CC	0,019	0,008						
	Russian	GG	0,808	0,750						
		GC	0,163	0,213	1,400	0,831-2,351	1,453	0,221	1,661	0,193
		CC	0,029	0,038						
rs13181	Kazakh	TT	0,192	0,202						
		GT	0,404	0,323	1,423	0,731-2,762	1,131	0,567	0,307	0,579
		GG	0,404	0,476						
	Russian	TT	0,295	0,265						
					0,856	0,561-1,301	0,599	0,741	0,092	0,761

Table S2. Allele and Genotype Frequency Distribution of Selected Genes in Individuals Associated with the Balkashinskoye (Shantobe)

**Polymorphism**	**Population Group**	**Genotype**	**Miners**	**Controls**	**OR (95% CI)**	**CI, (95%)**	**χ^2 (Genotype)**	**p (Genotype)**	**χ^2 (Allele)**	**p (Allele)**
APC	Kazakhs	CC	0,375	0,305						
		CT	0,500	0,508	0,969	0,343-2,741	0,061	0,970	0,095	0,757
		TT	0,125	0,188						
	Russians	CC	0,296	0,217						
		CT	0,481	0,522	0,851	0,448-1,619	1,244	0,536	0,207	0,988
		TT	0,222	0,261						
VEGF	Kazakh	DD	0,442	0,419						
		DI	0,365	0,426	0,969	0,532-1,771	0,012	0,912	0,842	0,941
		II	0,192	0,155						
	Russian	DD	0,285	0,272						
		DI	0,453	0,531	0,869	0,471-1,582	2,403	0,301	1,503	0,221
		II	0,262	0,198						
rs1801320	Kazakh	GG	0,865	0,822						
		GC	0,115	0,171	1,871	0,882-3,967	2,838	0,092	3,172	0,074
		CC	0,019	0,008						
	Russian	GG	0,808	0,750						
		GC	0,163	0,213	1,271	0,566-1,491	0,701	0,403	0,037	0,846
		CC	0,029	0,038						
rs13181	Kazakh	TT	0,018	0,202						
		GT	0,509	0,323	0,073	0,021-0,571	3,489	0,061	2,780	0,095
		GG	0,473	0,476						
	Russian	TT	0,205	0,281						
		GT	0,459	0,431	1,121	0,732-1,711	2,826	0,243	2,630	0,104
		GG	0,335	0,288						

https://doi.org/10.3390/genes17020191
